# An audit of Zimbabwean public sector diagnostic ultrasound services

**DOI:** 10.11604/pamj.2021.39.99.28342

**Published:** 2021-06-03

**Authors:** Humphrey Mapuranga, Richard Denys Pitcher, George Chanetsa Jakanani, Josephat Banhwa

**Affiliations:** 1 Division of Radiodiagnosis, Department of Medical Imaging and Clinical Oncology, Faculty of Medicine and Health Sciences, Stellenbosch University and Tygerberg Hospital, Cape Town, South Africa,; 2 Department of Radiology, Parirenyatwa Group of Hospitals, Harare, Zimbabwe,; 3 Department of Medical Physics and Imaging Sciences, Faculty of Medicine and Health Sciences, University of Zimbabwe, Harare, Zimbabwe

**Keywords:** Zimbabwe, universal health coverage, ultrasound, public sector, healthcare facility

## Abstract

**Introduction:**

the provision of basic diagnostic imaging services is pivotal to achieving universal health coverage. An estimated two-thirds of the world's population have no access to basic diagnostic imaging. Accurate data on current imaging equipment resources are required to inform health delivery strategy and policy at national level. This is an audit of Zimbabwean public sector diagnostic ultrasound resources and services.

**Methods:**

utilising the Ministry of Health and Child Care (MHCC) database, sequential interviews were conducted with provincial health authorities and local facility managers. Ultrasound equipment, personnel and services in all hospitals and clinics, nationally were recorded, collated, and analysed for the whole country, and by province.

**Results:**

of the 1798 Zimbabwean public sector healthcare facilities, sixty-six (n=66, 3.67%) have ultrasound equipment. Ninety-nine (n=99) ultrasound units are distributed across the sonar facilities, representing a national average of 8 units per million people. More than half the equipment units (n=53, 54%) are in secondary-level healthcare facilities (district and mission hospitals), and approximately one-fifth (n=22, 22%) in the central hospitals (quaternary level). The best-resourced province has twice the resources of the least resourced. One-hundred and forty-two (n=142) healthcare workers, from six different professional groups, provide the public sector ultrasound service. Most facilities with sonar equipment (n=64/66, 97%) provide obstetrics and gynaecology services, while general abdominal scanning is available at one third (n=22, 33%). Two facilities with ultrasound equipment have no capacity to offer a sonography service.

**Conclusion:**

in order to reach the WHO recommendation of 20 sonar units per million people, an estimated 140 additional sonar units are required nationally. The need is greatest in Masvingo, Midlands and Mashonaland East Provinces. Task-shifting plays a key role in the provision of Zimbabwean sonar services. Consideration should be given to formal training and accreditation of all healthcare workers involved in sonar service delivery.

## Introduction

A key goal of the United Nations (UN) 2030 Agenda for Sustainable Development is the achievement of universal health coverage (UHC), which may be defined as ensuring all people have access to effective health services, without financial hardship [1,2]. The WHO regards basic radiological services as essential in any healthcare system. Globally, radiology is fast assuming an integral role in diagnosis and management of patients [3-5]. The provision of plain X-ray and ultrasound services may thus be regarded as pivotal to the achievement of UHC. It is estimated that one X-ray and ultrasound unit for every 50000 people, or 20 units each per million people, will provide ninety percent of the global imaging requirements [6-8]. However, current estimates indicate that less than two-thirds of the world has access to any form of diagnostic imaging [9,10]. Resources amongst rural populations of low- and middle-income countries (LMICs) are particularly constrained [11].

In May 2007, the 60^th^ UN World Health Assembly adopted Resolution 60.29, urging member states to “*collect, verify, update and exchange information on health technologies, in particular medical devices, as an aid to their prioritization of needs and allocation of resources*” [12]. However, LMIC radiological needs are poorly documented. Although the WHO has published national estimates of high-end medical imaging equipment, such as computed tomography (CT) and magnetic resonance (MR) scanners, based on questionnaire surveys of member countries, data on basic diagnostic imaging equipment such as plain X-ray and ultrasound are not included [9]. Governments require accurate data on current imaging equipment to inform, shape and guide national strategy and policy. Resource disparities can only be redressed if such data are available. It is in this context that the Division of Radiodiagnosis of the Department of Medical Imaging and Clinical Oncology at Stellenbosch University has embarked on a systematic, collaborative evaluation of diagnostic radiology resources in African countries, with a view to providing reference data for healthcare planning. To date, analyses of registered imaging equipment resources have been completed for South Africa, Tanzania, Zimbabwe, Zambia and Uganda [6-8,13,14].

However, these analyses only include resources registered with national regulatory authorities. Since ultrasound does not produce ionizing radiation, registration of ultrasound equipment is not required. There thus exists no national registry of ultrasound equipment for any country, worldwide. This constitutes a major drawback in the appraisal of national imaging capacity, especially in LMICs, where ultrasound has the potential to serve a key role in the healthcare system [3]. Ultrasound has many characteristics making it uniquely suited to the provision of healthcare in less resourced environments. It is relatively affordable and generates no ionizing radiation; it is therefore safe and has no specific installation or radiation protection requirements. It operates on standard electrical supply but may also be battery operated. Machines are robust, mobile, potentially portable and require relatively little maintenance. The smallest units fit into the palm of a hand. Sonar can be used repeatedly on the same patient, posing no threat to the unborn child, making it ideal for antenatal imaging. It provides excellent imaging of the soft tissues, and thus satisfactory first-line imaging of the abdomen, pelvis, heart, muscles, tendons, and the soft tissues of the neck. Its main use in the chest is the detection and characterisation of pleural effusions. Ultrasound can guide chest drain insertion, minor interventional procedures such as abscess drainage, as well as tissue biopsy. Doppler ultrasound affords exceptional vascular imaging. With appropriate training, sonar can be utilized by the broad spectrum of medical personnel. Competence can be on a sliding scale, from basic to highly sophisticated, with incremental improvement over-time. Ultrasound generates real-time, high-resolution digital images, which can be interpreted during the examination, while captured images and video clips may also be stored in a digital archive and transmitted via the internet for expert remote reporting [3]. The aim of this study was to conduct the first national audit of a public sector diagnostic ultrasound service.

## Methods

The study was conducted in Zimbabwe, a landlocked Southern African country with a total land area of 390757 square kilometres, a population of 13.06 million people and 10 administrative provinces [15]. Zimbabwe's Primary Healthcare System includes primary level care provided at clinics and rural health centres, secondary care at district hospitals, tertiary care at general and provincial hospitals, and quaternary care at central teaching hospitals. Mission hospitals are accessible to all, make a substantial contribution to rural secondary-level services, may be considered equivalent to district hospitals, and were thus included in this analysis of public sector services. In 2015, maternal mortality was 651/10^5^ live births, while under-five mortality was 69/10^3^ live births. The HIV prevalence rate for the population 15-49 years was 13.8 percent [15].

In 2015, Zimbabwe's national per capita healthcare expenditure was 25 USD. While lower than the WHO recommendation of 86USD per capita, more than half the annual national health funding (56%) is from external donors [11]. Approximately ten percent of the population has access to private healthcare. The Zimbabwean Ministry of Health and Child Care (MHCC), and the University of Zimbabwe's Department of Medical Physics and Imaging Sciences were key collaborators in this work. Since no formal government policy exists with respect to the provision of sonar services, phase 1 of the project involved contacting the ten provincial health authorities for an overview of sonar services at provincial level, and identification of all provincial facilities with sonar equipment. Phase 2 involved interrogation of the MHCC database for the contact details of all facilities identified in Phase 1 as having sonar equipment. In phase 3, the managers of all facilities with sonar equipment cooperated in a telephonic survey on their institution's ultrasound equipment, staff complement, staff expertise and services. Survey data were captured on a customised spreadsheet and analysed for the whole country, and by province. Descriptive statistics defined resources per 1000 square kilometres and per million people. The study was approved by the Health Research Ethics Committee of the Faculty of Medicine and Health Sciences of Stellenbosch University, Cape Town, South Africa and by the MHCC of Zimbabwe through the Medical Research Council of Zimbabwe.

## Results

### National analysis

**Facilities:** of the 1798 Zimbabwean public sector healthcare facilities, sixty-six (n = 66, 3.7%) have ultrasound equipment, equating to a national average of 6 facilities/10^6^ people and 1 facility per 5000 square kilometres. Although all levels of healthcare facility may provide sonographic services, secondary level institutions in the form of district (n = 32/66, 48.5%) and mission hospitals (n = 12/66, 18.2%) together constitute two-thirds (n = 44, 67%) of all such facilities (Table 1).

**Table 1:** analysis by health-care facility

Health-care facility	All facilities	Facilities with ultrasound equipment	No. of ultrasound units.	Health care workers n (%)
Central hospitals	6(0.3%)	5(7.6%)	22(22.2%)	69(48.6%)
Provincial hospitals	7(0.4%)	7(10.6%)	14(14.2%)	23(16.2%)
General hospitals	5(0.3%)	5(7.6%)	5(5.1%)	6 (4.2%)
District hospitals	45(2.5%)	32(48.5%)	41(41.4%)	33(23.2%)
Mission hospitals	56(3.1%)	12(18.2%)	12(12.1%)	8(5.6%)
Rural health hospitals	77(4.3%)	2(3.0%)	2(2.0%)	2(1.5%)
Clinics	1602(89.1%)	3(4.5%)	3(3.03%)	1(0.7%)
**Totals**	**1798(100%)**	**66(100%)**	**99(100%)**	**142(100%)**

**Equipment:** ninety-nine (n = 99) ultrasound units are distributed between the sonar facilities, representing a national average of 8 units/10^6^ people. More than half the units (n = 53, 54%) are in district and mission hospitals, while approximately one-fifth (n = 22, 22%) are in the central hospitals (Table 1).

**Health care workers (HCW):** one-hundred and forty-two (n = 142) HCW, from six different professional groupings, provide services (Table 2).

**Table 2:** analysis by health-care workers

Healthcare workers	Central hospital	Provincial hospital	General hospital	District hospital	Mission hospital	Rural hospital	Municipal clinic	Total
Radiographer	41	14	4	5	0	0	1	65
Medical officer	0	2	1	6	4	2	0	15
Medical specialists	15	3	0	0	0	0	0	18
Sonographer	8	3	0	0	0	0	0	11
Midwife	5	0	0	6	0	0	0	11
X-ray operator	0	1	1	16	4	0	0	22
**Total**	**69**	**23**	**6**	**33**	**8**	**2**	**1**	**142**

**a) Medical specialists (n = 18, 13%):** specialist training across numerous disciplines includes ultrasound instruction, with the acquisition of speciality-specific scanning proficiency. There is no formal accreditation of ultrasound expertise. One consultant radiologist in Harare undertakes in-patient abdominal, pelvic, chest, musculoskeletal, cardiac and neonatal examinations on referral from sonographers, medical officers and medical specialists. A single foetal medicine specialist in Harare performs general antenatal, as well as sub-specialist-level anomaly scanning and management. Thirteen obstetricians and gynaecologists in the three central hospitals and one in the Mutare Provincial Hospital deliver general obstetrics and gynaecology scanning services. One specialist physician and one paediatrician in Mutare Provincial Hospital provide echocardiography services.

**b) Medical officers (n=15, 11%):** fifteen medical officers across the country have acquired ultrasound skills through in-house and continuous education training programs. There exists no limit or regulation to the scope of practice and no formal ultrasound accreditation or qualification is conferred.

**c) Sonographers (n=11, 8%):** two Zimbabwean institutions conduct accredited ultrasound training for qualified radiographers. The National University of Science and Technology offers a 24-month Master of Science program, and the Harare Institute of Technology conducts an 18-month postgraduate diploma. Additionally, the Burwin Institute of Diagnostic Medical Ultrasound, in Winnipeg, Canada, offers home-study theoretical modules accredited by the Allied Health Practitioners Council of Zimbabwe. Upon completing the required theoretical courses, candidates are required to undertake a 6-month supervised attachment at any local recognized health facility. Radiographers successfully completing any of the above three programs acquire formal sonographer accreditation, with scope of practise across all organ systems.

**d) Radiographers (n = 65, 46%):** two institutions, the National University of Science and Technology, and the Zimbabwe School of Radiography, affiliated to the University of Zimbabwe College of Health Sciences, offer a four-year degree programme in radiography. Prospective candidates must achieve at least two advanced-level passes in biology, mathematics, physics or chemistry of the General Certificate of Education from an approved board. A three-year radiography diploma program, with similar entry requirements, previously conducted by the school of radiography, was replaced by the degree program in 2018. Both graduates and diploma holders are accredited by the Allied Health Practitioners Council of Zimbabwe to perform obstetrics and gynaecology ultrasonography. Additionally, qualified sonographers conduct informal, in-house radiographer training programs in general abdominal ultrasound, to expand the scope of basic services in underserved rural populations. Such training is not formally accredited.

**e) Midwives (n = 11, 8%):** the Zimbabwe School of Midwifery, of the MHCC, enrols students for a 1-year diploma. Prospective candidates are required to be either state-certified or state-registered nurses, with a minimum of two years' experience. The diploma is accredited by the Nurses Council of Zimbabwe. Provincial-based midwives undergo informal training in basic obstetric ultrasound, conducted by provincial sonographers. The training is demand- and needs-driven and is aimed at addressing the obstetric service challenges in underserved populations. No specific course duration or formal accreditation is accorded this training.

**f) X-ray operators (n = 22, 15%):** X-ray operators are trained by the Zimbabwe School of Radiography. On completion of the 18-month course, a certificate in basic radiography is conferred, accredited by the Allied Health Practitioners Council of Zimbabwe. Candidates are required to have Ordinary-level passes of the General Certificate of Education from an approved board. X-ray operators are principally stationed in district hospitals and a have undergone needs-driven in-house basic training by provincial sonographers. There is no specific course duration or formal accreditation for this training.

**Overview:** radiographers (n = 65/142, 46%) constitute the largest professional cohort providing sonographic services. Almost half the sonographic workforce (n = 69, 49%) is in the five central teaching hospitals in Harare and Bulawayo, including all sonographers (n = 11/11, 100%) and more than eighty percent of radiographers (n = 55/65, 85%). Most X-ray operators (n = 20/22, 91%) are deployed in district or mission hospitals. Amongst the ultrasound workforce, only sonographers (n = 11/142, 8%), have formal accreditation of sonographic training. By virtue of their specialisation, the diagnostic radiologist, foetal medicine specialist and obstetricians and gynaecologists do not require separate accreditation to perform sonography.

**Services:** most facilities, (n = 64/66; 97%) offer obstetrics and gynaecology services, while general abdominal scanning is available at one third (n = 22/66, 33%) of facilities. Limited echocardiography is performed in Harare and Manicaland. Neonatal services are confined to Harare. There are two facilities with equipment but no skilled human capacity to provide sonography services.

### Provincial analysis

**Facilities:** the most sparsely populated provinces of Matabeleland North and Matabeleland South have the highest number of sonar facilities/10^6^ people, with 9 and 10 facilities respectively (Table 3). Conversely, Harare and Bulawayo, with the highest population density, have the lowest resources by population, each with 3 facilities/10^6^ people. However, Harare has the most geographically accessible services, with 6.9 facilities per thousand square kilometres, being 89 times more accessible than those of Matabeleland North (0.08 facilities/10^3^ km^2^).

**Table 3:** Zimbabwe public sector diagnostic ultrasound services by population province, sonar units and healthcare workers per million people

Province					Facilities with sonar units					Units				Health care workers(n)							Services			
**Name**	**Land area (km^2^)**	**Population dependant on PHC (x 10^6^)**	**Total population**	**Pop. density (people/km^2^)**	**HCF**	**(n)**	**tot**	**/10^3^ km^2^**	**/10^6^ people**	**(n)**	**tot**	**/10^6^ people**	**R**	**Mo**	**Ms**	**So**	**Mw**	**Xo**	**total**	**HCW/10^6^ people**	**G**	**O&G**	**Neo**	**Echo**
**Harare**	872	1,910 818	2,123,132	2,406	**Cent. H**	3	6	6.9	3	14	17	9	25	-	*10	7	5	0	48	25.1	X	X	X	X
					**Muni clin**	3				3			1	0	-	0	0	0			0	X	0	0
**Bulawayo**	479	588,004	653,337	1,369	**Cent. H**	2	2	4.2	3	8	8	14	16	-	**5	1	0	0	22	37.4	X	X	0	0
**Matabeleland North**	75,025	674,115	749,017	10	**District H**	5	6	0.08	9	7	8	12	1	1	-	0	0	4	7	10.4	0	X	0	0
					**Mission H**	1				1			0	0	-	0	0	1			0	X	0	0
**Matabeleland South**	54,172	615 504	683,893	13	**Province H**	1	6	0.11	10	2	7	11	2	1	-	0	0	1	7	11.4	X	X	0	0
					**District H**	5				5			1	1	-	0	1	0			X	X	0	0
**Masvingo**	56 566	1,336,581	1,485,090	26	**Province H**	1	9	0.16	6.7	1	9	7	2	0	-	0	0	0	6	4.5	0	X	0	0
					**District H**	4				4			0	2	-	0	0	0			0	X	0	0
					**Mission H**	4				4			0	2	-	0	0	0			0	X	0	0
**Mashonaland East**	32 230	1,210,460	1,344,955	41	**Province H**	1	6	0.19	4.9	2	8	7	1	0	-	0	0	0	6	5	0	X	0	0
					**General H**	1				1			1	0	-	0	0	0			0	X	0	0
					**District H**	3				4			0	1	-	0	0	2			0	X	0	0
					**Mission H**	1				1			0	0	-	0	0	1			0	X	0	0
**Mashonaland West**	57 441	1,351,490	1,501,656	25	**Province H**	1	7	0.12	5.2	2	12	9	3	0	-	1	0	0	15	11.1	X	X	0	0
					**General H**	1				1			1	0	-	0	0	1			X	X	0	0
					**District H**	4				8			1	0	-	0	3	4			0	0	0	0
					**Mission H**	1				1			0	0	-	0	0	1			0	0	0	0
**Manicaland**	36,459	1,577,428	1,752,698	48	**Province H**	1	8	0.22	5.1	2	11	7	2	-	∞3	1	0	0	15	9.5	X	X	0	X
					**General H**	1				1			1	0	-	0	0	0			X	X	0	0
					**District H**	4				6			2	0	-	0	2	3			0	X	0	0
					**Mission H**	2				2			0	0	-	0	0	1			0	X	0	0
**Mashonaland Central**	28,347	1,037,268	1,152,520	40	**Province H**	1	7	0.25	6.7	3	9	9	0	1	-	1	0	0	7	6.7	X	X	0	0
					**General H**	1				1			0	1	-	0	0	0			X	X	0	0
					**District H**	2				2			0	0	-	0	0	2			0	X	0	0
					**Mission H**	3				3			0	2	-	0	0	0			0	X	0	0
**Midlands**	49,166	1,453,447	1,614,941	33	**Province H**	1	9	0.18	6.2	2	10	7	4	0	-	0	0	0	9	6.2	0	X	0	0
					**General H**	1				1			1	0	-	0	0	0			0	X	0	0
					**District H**	5				5			0	1	-	0	0	1			X	X	0	0
					**Rural H**	2				2			0	2	-	0	0	0			0	X	0	0
**Total**	**390,757**	**11,755,115**	**13,061,239**				**66**	**0.17**	**5.6**		**99**	**8.4**	**65**	**15**	**18**	**11**	**11**	**22**	**142**	**12.07**				

R - Radiographer, Mo - Medical officer, Ms - Medical specialist, So - Ultrasonographer, Mw - Midwife, Xo - X ray operator, HCF - Healthcare facility, Neo - Neonatology, Echo - Echocardiography, O&G - Obstetrics & gynaecology, G - General

**Equipment:** Bulawayo, with 14 units/10^6^ people, is the best equipped province by population, having twice the resources of the four least resourced provinces of Mashonaland East, Manicaland, Masvingo and the Midlands, each with 7 units/10^6^ people.

**Health care workers:** all provinces have an ultrasound workforce which includes at least one radiographer or sonographer. Bulawayo, with sixteen percent of the ultrasound workforce (n = 22, 15.5%) and 37 HCW/10^6^ people is the best resourced by population, having just over 8 times the resources of Masvingo, the least resourced, with just over 4.2% of the workforce, equating to 4.5 HCW/10^6^ people (n = 6, 4.2%; 4/10^6^ people). Harare and Bulawayo have the highest number of HCW per equipment unit (2.9), while Mashonaland Central has the lowest (0.6).

**Services:** all provinces offer basic obstetrics and gynaecological services at provincial and/or district hospitals. Seven provinces (7/10, 70%) offer abdominal services. Matabeleland North, Masvingo and Mashonaland East, with a combined population of 3.2 million people, or more than a quarter (27.2%) of the population dependant on public sector services, have no direct access to abdominal sonar services. Only Harare has the full range of ultrasound services.

## Discussion

This is the first national audit of public-sector ultrasound resources and services in any healthcare setting. It thus makes a seminal contribution to global health services research. Furthermore, the work is closely aligned with UN Resolution 60.29 of 2007 which explicitly calls for the collation of national medical imaging resource data, to optimise equipment allocation. As such, it has the potential to contribute to the achievement of universal health coverage, a key health target of the UN 2030 Sustainable Development Goals (SDGs) [1]. Zimbabwe's 8 sonar units/10^6^ people are less than half the WHO recommendation of 20 units/10^6^ people, reflecting a shortfall of 12 units/10^6^ people. Given the estimated 11.8 million Zimbabweans reliant on public health services, this equates to an absolute national deficit of approximately 140 units. A mid-range ultrasound machine, with full functionality, costs US$15,000 - USD$20,000. Approximately 2-3 million USD would thus be required to align sonar equipment resources with WHO recommendations. However, the cost of sonar equipment is decreasing and small, hand-held units are becoming progressively more affordable. Thus, enhancing sonar resources nationally need not be prohibitively expensive. These data also provide key insights into the appropriate allocation of additional units, highlighting the greatest need in Masvingo, the Midlands, and Mashonaland East provinces.

The finding that obstetrics and gynaecology scanning is Zimbabwe's most widely available sonar service is consistent with previous reports on LMIC sonar practices [16-18] which showed that pregnancy-related conditions are the main disease burden at district hospitals [19]. This study provides important insights into the role of task-shifting in the provision of Zimbabwean antenatal sonar services, showing that almost a quarter (33/142, 23%) of service providers, namely midwives (n =11) and X-ray operators (n =22), have only informal, in-house training. Task-shifting in the provision of basic obstetric ultrasound services has been documented in several resource-constrained settings, and is increasingly invoked, globally. Moreover, there is considerable published work on the success of short, basic antenatal sonar training programs for rural health workers [20-23]. The Zimbabwean model for basic obstetric scanning is thus in keeping with international trends in resource-limited environments. This study affords the most detailed published data to date on the role of task-shifting in antenatal sonar service at national level, demonstrating how a tiered system of HCW utilization has evolved in response to service pressures at the various levels of health care. Thought could thus be given to formal training and accreditation of the respective levels of expertise of the various HCWs, specifically midwives and X-ray operators.

Basic obstetric sonar services can play a key role in reducing maternal and perinatal mortality. Of note, the UN 2030 Agenda for Sustainable Development aims to decrease global maternal mortality to less than 70/10^5^ live births and neonatal mortality to 12/10^3^ live births [1]. Most maternal deaths are preventable, since methods for managing the complications of pregnancy and childbirth are well known. Maternal death is mainly caused by haemorrhage, sepsis, hypertension in pregnancy and the interaction of pregnancy with pre-existing medical conditions. Antenatal ultrasound confirms intra-uterine pregnancy, accurately estimates gestational age, predicts the expected delivery date, and facilitates planned, term delivery at an appropriate facility. Antenatal ultrasound also identifies the “at-risk” pregnancy, most notably ectopic/multiple pregnancy, foetal anomaly and abnormal placental position, allowing appropriate referral and intervention. During labour, ultrasound demonstrates abnormal foetal presentation and obstructed labour, prompting early intervention. After delivery, ultrasound identifies retained products of conception. Thus, by demonstrating conditions predisposing to haemorrhage, such as ectopic pregnancy, abnormal placental position, obstructed labour and retained intrauterine products, ultrasound addresses one of the main causes of maternal mortality. Similarly, early identification of retained products facilitates timely uterine evacuation, limiting the risk of puerperal sepsis.

Antenatal ultrasound also plays an important role in addressing birth asphyxia. Through accurate gestational age estimation, ultrasound facilitates planned delivery, at term, at an appropriate healthcare facility. Additionally, through the monitoring of foetal growth and amniotic fluid volume, ultrasound assesses foetal well-being, facilitating referral at the earliest sign of growth retardation or placental insufficiency. During delivery, ultrasound accurately demonstrates complications of labour, facilitating early intervention. The absence of an abdominal sonar service in three of ten provinces, and in two-thirds of facilities providing an obstetric sonar service is noteworthy. It is now three decades since the pioneering prospective work of Mets, highlighting the potential contribution of abdominal sonography to clinical management in elderly patients in resource-limited environments, with sonographic findings contributing to problem-solving in seventy percent of cases [24]. More recently, Shah and co-workers showed that abdominal investigations constituted more than forty percent of the workload of a newly introduced sonar service in rural Uganda and that sonar findings influenced patient management in almost half the cases [25]. Of note, all Zimbabwean provincial, district and mission hospitals are staffed by medical officers, who could be earmarked for abdominal sonar training courses. A limitation of this study was that quality assurance of imaging equipment and personnel was not included in the methodology. Additionally, the number of studies performed at the respective facilities could not be quantified. It is recommended that future studies include these aspects. It is hoped that this work will stimulate further, similar national audits of sonar services worldwide.

## Conclusion

Zimbabwe has an estimated shortfall of 12 sonar units/10^6^ people. In order to reach the WHO recommendation of 20 units/10^6^ people, a further 140 sonar units are required nationally. The need is greatest in Masvingo, Midlands and Mashonaland East Province. Task-shifting plays a key role in the provision of Zimbabwean sonar services. Consideration should be given to formal training and accreditation of all healthcare workers involved in sonar service delivery.

### What is known about this topic

- Less than two thirds of the world has access to basic diagnostic imaging services. Basic radiological services amongst rural populations of low-and middle-income countries (LMICS) are particularly constrained;
- There exists no national registry of ultrasound equipment for any country, worldwide. This constitutes a major drawback in the appraisal of national imaging capacity, especially in LMICs, where ultrasound has the potential to serve a pivotal role in the healthcare system.

### What this study adds

- This is the first national audit of public-sector ultrasound resources and services in any healthcare setting. It therefore makes a seminal contribution to global health services research;
- The study is directly aligned with UN Resolution 60.29 of 2007 which calls for the collation of national medical imaging resource data to optimise equipment allocation. It offers great potential to contribute to the achievement of universal health coverage, a key health target of the UN 2030 Sustainable Development Goals (SDGs);
- This work represents the most detailed published data to date on the role of task-shifting in antenatal sonar service at national level, highlighting how a tiered system of HCW utilization has evolved in response to service pressures. Ultrasound skills training within the various healthcare tiers can be used effectively to improve service delivery nationally.

## References

[1] United Nations Transforming our World. The 2030 agenda for sustainable development A/RES/70/.

[2] (2017). Tracking universal health coverage: 2017 global monitoring report. World Health Organisation and International Bank for Reconstruction and Development/ The World Bank.

[3] Pitcher RD, Mollura D, Culp M, Lungren M (2019). The role of radiology in global health. Radiology in Global Health.

[4] World Health Organisation (1978). Declaration of Alma-Ata; International Conference on Primary Health Care. Alma-Ata USSR.

[5] Palmer PES (1978). Radiology and primary care. Pan American Health Organisation.

[6] Kabongo JM, Nel S, Pitcher RD (2015). Analysis of licensed South African diagnostic imaging equipment. Pan Afr Med J.

[7] Ngoya PS, Muhogora WE, Pitcher RD (2016). Defining the diagnostic divide: an analysis of registered radiological equipment resources in a low-income African country. Pan Afr Med J.

[8] Maboreke T, Banhwa J, Pitcher RD (2019). An audit of licensed zimbabwean radiology equipment resources as measure of healthcare access and equity. Pan Afr Med J.

[9] World Health Organization (2010). Baseline country survey on medical devices.

[10] World Health Organisation (2017). Global atlas of medical devices. WHO medical devices series ISBN 978-924-151231-2.

[11] World Health Organisation Global health expenditure database.

[12] World Health Organisation Sixteeth World Health Assembly.

[13] Mbewe C, Chanda-Kapata P, Sunkutu-Sichizya V, Lambwe N, Yakovlyeva N, Chirwa M (2020). An audit of licenced Zambian diagnostic imaging equipment and personnel. Pan Afr Med J.

[14] Kiguli-Malwadde E, Byanyima R, Kawooya MG, Mubuuke AG, Basiimwa RC, Pitcher R (2020). An audit of registered Ugandan radiology equipment resources. Pan Afr Med J.

[15] Zimbabwe National Statistics Agency-ZIMSTAT (2016). Zimbabwe Demographic and Health Survey.

[16] Groen RS, Leow JJ, Sadasivam V, Kushner AL (2011). Review: indications for ultrasound use in low-and middle-income countries. Trop Med Int Health.

[17] Kim ET, Singh K, Moran A, Armbruster D, Kozuki N (2018). Obstetric ultrasound use in low and middle income countries: a narrative review. Reprod Health.

[18] Sippel S, Muruganandan K, Levine A, Shah S (2011). Review article: use of ultrasound in the developing world. Int J Emerg Med.

[19] Bussmann H, Koen E, Arhin-Tenkorang D, Munyadzwe G, Troeger J (2001). Feasibility of an ultrasound service on district health care level in Botswana. Trop Med Int Health.

[20] Greenwold N, Wallace S, Prost A, Jauniaux E (2014). Implementing an obstetric ultrasound training program in rural Africa. Int J Gynaecol Obstet.

[21] Kozuki N, Mullany LC, Khatry SK, Ghimire RK, Paudel S, Blakemore K (2016). Accuracy of home-based ultrasonographic diagnosis of obstetric risk factors by primary-level health care workers in rural nepal. Obstet Gynecol.

[22] Swanson D, Lokangaka A, Bauserman M, Swanson J, Nathan RO, Tshefu A (2017). Challenges of implementing antenatal ultrasound screening in a rural study site: a case study from the Democratic Republic of the Congo. Glob Health Sci Pract.

[23] Awor P, Nabiryo M, Manderson L (2020). Innovations in maternal and child health: case studies from Uganda. Infect Dis Poverty.

[24] Mets T (1991). Clinical ultrasound in developing countries. Lancet.

[25] Shah SP, Epino H, Bukhman G, Umulisa I, Dushimiyimana JMV, Reichman A (2009). Impact of the introduction of ultrasound services in a limited resource setting: rural Rwanda 2008. BMC Int Health Hum Rights.

